# Comparative Analysis of Bilateral Open Inguinal Hernia Repair and Rives-Stoppa Repair: A Comprehensive Review

**DOI:** 10.7759/cureus.57431

**Published:** 2024-04-01

**Authors:** Yashraj Jain, Sanjeev G Gianchandani Gyani, Simran Chauhan, Krushank Nayak, Yuvraj Jain, Geetika Malhotra, Sai Goutham Rekavari

**Affiliations:** 1 Surgery, Rajshree Nursing Home, Ashoknagar, IND; 2 Minimal Access And Robotic Surgery, Anglia Ruskin University, Chelmsford, GBR; 3 Surgery, Jawaharlal Nehru Medical College, Datta Meghe Institute of Higher Education and Research, Wardha, IND; 4 Surgery, Bharti vidyapeeth medical college and hospital, Sangli, IND

**Keywords:** patient outcomes, comparative analysis, rives-stoppa, bilateral repair, surgical techniques, inguinal hernias

## Abstract

Inguinal hernias present a significant healthcare burden globally, necessitating effective surgical management. This comprehensive review evaluates two primary surgical techniques for managing bilateral inguinal hernias: bilateral open inguinal hernia and Rives-Stoppa repair. This review aims to provide insights into optimal surgical approaches through a comparative analysis of these techniques, including examining advantages, disadvantages, outcomes, and factors influencing technique selection. Bilateral open inguinal hernia repair offers simplicity and familiarity, while Rives-Stoppa repair may provide advantages such as reduced recurrence rates and postoperative complications. The findings underscore the importance of considering patient-specific factors, surgeon expertise, and hospital resources when selecting the optimal approach. Further research is warranted to conduct long-term comparative studies and explore innovations in surgical techniques and materials, ultimately enhancing patient outcomes and advancing inguinal hernia repair practices.

## Introduction and background

Inguinal hernias represent a prevalent surgical condition encountered globally, affecting millions of individuals annually. These hernias occur when abdominal organs, such as the intestines or fatty tissue, protrude through weakened or torn areas in the inguinal canal - a crucial passage in the lower abdomen [1]. The causes of inguinal hernias vary, including congenital predisposition, age-related weakening of abdominal tissues, heavy lifting, chronic coughing, or obesity. They can manifest unilaterally, affecting one side of the groin or bilaterally, involving both sides. Moreover, their incidence tends to rise with age and is notably more common in males. Left untreated, inguinal hernias can lead to severe complications such as bowel obstruction or strangulation, which necessitate emergency surgical intervention to prevent life-threatening consequences [2].

Effective management of inguinal hernias hinges upon selecting appropriate hernia repair techniques. The choice of surgical approach plays a pivotal role in determining patient outcomes, encompassing factors such as postoperative pain levels, recovery duration, and the risk of hernia recurrence [3]. Surgeons have various options, including open repair techniques like traditional tension-free mesh repair, the Rives-Stoppa technique, and minimally invasive approaches such as laparoscopic hernia repair. Each technique boasts its own advantages and disadvantages, with selection contingent on factors like hernia size, patient comorbidities, surgeon expertise, and patient preferences. The optimal choice of technique is paramount for achieving successful outcomes and ensuring patient satisfaction [4].

The primary objective of this review is to conduct a thorough comparative analysis of two frequently utilized surgical techniques for managing bilateral inguinal hernias: bilateral open inguinal hernia repair and Rives-Stoppa repair. By meticulously examining the advantages, disadvantages, outcomes, and factors influencing technique selection for each approach, this review aims to provide valuable insights to healthcare professionals and surgeons. Through synthesizing existing evidence, this review endeavors to facilitate informed decision-making regarding the optimal surgical approach for managing bilateral inguinal hernias, ultimately enhancing patient care and outcomes. Such an analysis is crucial in navigating the complexities of inguinal hernia management and ensuring that patients receive the most appropriate and effective treatment tailored to their needs and circumstances.

## Review

Bilateral open inguinal hernia repair

Definition and Overview

Hernia repair entails a series of surgical procedures, including the isolation and dissection of the hernia sac, the reduction of intraperitoneal contents, fascial repair, and the closure of soft tissues. In 1993, the American College of Surgeons (ACS) proposed a code change to the American Medical Association CPT Editorial Panel to revise hernia coding. Consequently, codes for open repair of inguinal and umbilical hernias were incorporated. These codes were subsequently categorized based on whether the hernias were reducible or incarcerated/strangulated, except for rare lumbar or spigelian hernia repairs [5]. Notably, hernia repair codes are not contingent on the size of the hernia repaired. This implies that regardless of operative time and effort variations, repairs of different hernia sizes receive the same payment. Moreover, when multiple hernias are repaired during the same operative session and through the same incision, they cannot be coded separately [6].

Surgical Technique

The surgical approach for open inguinal hernia repair encompasses several techniques, including herniotomy, herniorrhaphy, and hernioplasty. One prominent example of hernioplasty is the Lichtenstein tension-free mesh repair, which reinforces the weakened posterior wall with mesh without directly repairing it [7]. In open anterior repair methods such as Bassini, McVay, and Shouldice repairs, the external oblique aponeurosis is incised to release the spermatic cord and reconstruct the inguinal canal floor using permanent sutures [8]. Conversely, posterior repair techniques like iliopubic tract repair and the Nyhus technique involve dissection behind and deep into the inguinal region to reconstruct from the *inside* [8].

Alternatively, laparoscopic repair presents another option for inguinal hernia repair, where specialized instruments are inserted through small abdominal incisions, and mesh is used for reinforcement. Compared to open surgery, laparoscopic repair offers a lower recurrence rate and faster recovery [9,10]. The choice between open and laparoscopic techniques depends on factors such as overall health status, surgeon expertise, and specific hernia characteristics [10].

Advantages

Open inguinal hernia repair offers several advantages, including its effectiveness in addressing the hernia, preventing serious complications, and facilitating quicker patient return to normal activities [11]. Techniques like the Lichtenstein tension-free mesh repair, a common open repair method, involve reinforcing the posterior wall of the inguinal canal with synthetic mesh, resulting in favorable outcomes [12]. Moreover, open repair procedures are generally safe and can be conducted under local anesthesia, making them preferable for many patients [12]. However, while open repair presents specific benefits, laparoscopic repair is linked with less postoperative pain and earlier resumption of everyday activities and work compared to its open counterpart [13]. Ultimately, the choice between open and laparoscopic approaches should consider factors such as pain management, recovery duration, cost considerations, and anesthesia preferences tailored to each patient's needs and goals [13].

Disadvantages

The disadvantages of open inguinal hernia repair encompass several significant concerns for patients undergoing this procedure. One notable drawback is the risk of chronic postoperative pain, which can pose a considerable challenge for individuals [7]. Moreover, complications such as infection, bleeding, and seroma formation at the incision site are commonly associated with hernia surgery [14]. Furthermore, there are long-term risks associated with open hernia repair, including chronic groin pain, issues with mesh movement or breakdown, and the potential for hernia recurrence [14]. Additionally, open surgery carries rare but severe risks, such as injury to blood vessels and nearby organs, complications related to anesthesia, and, in extreme cases, even death [14]. Given these considerations, patients must engage in thorough discussions with their healthcare providers regarding the benefits and risks of open inguinal hernia repair before deciding on the most suitable treatment approach [10].

Outcomes and Success Rates

The outcomes and success rates of inguinal hernia repair can vary depending on the chosen technique. Studies have demonstrated that laparoscopic repair, such as the TEP approach utilizing self-fixation mesh, represents an excellent option for inguinal hernia repair, with satisfactory results observed during follow-up and minimal complications reported [15]. Additionally, findings from a cohort study comparing mesh inguinal hernia repair performed by medical doctors and surgeons in Ghana revealed no significant difference in hernia recurrence rates at the one-year mark, suggesting that medical doctors can effectively undergo training to execute this type of repair [16]. Moreover, while laparoscopic repair offers advantages such as reduced postoperative pain and expedited recovery, open repair techniques have also exhibited notable success rates. The Stoppa procedure, in particular, is considered safe for addressing bilateral inguinal hernias, showing advancements over time in terms of operative duration, hospital stay duration, and morbidity rates [17].

Rives-Stoppa repair

Definition and Overview

The Rives-Stoppa repair, also known as the giant prosthetic reinforcement of the visceral sac (GPRVS), is a surgical technique designed to achieve anatomical and prosthetic repair by reinforcing the Myo pectineal region. This method is particularly well-suited for addressing complex inguinal hernias, especially recurrence cases or large and bilateral inguinoscrotal hernias. It entails a thorough dissection of the subfascial preperitoneal (PP) space and is typically performed on patients deemed suitable for general anesthesia. The utilization of mesh in this procedure contributes to a physiological healing process and facilitates bilateral anatomical reinforcement, rendering it a suitable option for challenging hernia cases [18]. Conversely, laparoscopic inguinal hernia repair techniques, such as the transabdominal preperitoneal (TAPP) or TEP approaches, share similar indications with open repair methods. These laparoscopic techniques are advantageous for addressing bilateral inguinal hernias and recurrent cases resulting from anterior approaches. They may offer benefits such as reduced postoperative pain and an earlier return to activity, particularly for specific demographics like young, active males with primary hernias. However, prior lower abdominal surgery or pelvic radiation can pose relative contraindications due to potential challenges in accessing the PP space [19].

Surgical Technique

The Rives-Stoppa repair technique entails dissecting a retro muscular plane between the muscle bellies and the posterior aponeurosis of the abdominal rectus muscles. This dissection allows for a tension-free closure of the musculoaponeurotic flap in the midline, effectively reconstructing the anatomy of the abdominal wall [20]. Particularly advantageous for addressing complex incisional hernias, this method offers lower recurrence rates and reduces complications by preventing direct mesh contact with the bowel. This prevention, in turn, decreases intrabdominal adhesions and facilitates future reoperations if necessary [21,22]. The procedural steps typically involve opening the hernia sac, performing adhesiolysis, making a longitudinal incision on the posterior sheath of the rectus muscle, dividing the retro muscular space, closing the posterior sheath, placing mesh in the retro muscular space, and finally achieving tension-free closure of the anterior musculoaponeurotic flap to reconstruct the midline [20].

Advantages

The Rives-Stoppa repair technique stands out for its numerous advantages in addressing complex incisional hernias. Extensive studies have consistently demonstrated that this method yields excellent long-term outcomes with remarkably low morbidity rates, particularly in patients with large primary or recurrent incisional hernias. This establishes it as the gold standard for many surgeons [23]. Characterized by a retromuscular approach, the Rives-Stoppa procedure has proven highly effective, boasting a high likelihood of achieving the lowest odds for both recurrence and surgical site infections (SSI) [23]. Moreover, a modified version of the Rives-Stoppa repair has notably reduced recurrence rates and complications, with only a minimal proportion of patients experiencing recurrent incisional hernias [23]. Comparative analyses have indicated that when contrasted with onlay mesh repair techniques, such as the retro-rectus (Rives-Stoppa) repair, the Rives-Stoppa technique showcases favorable outcomes, often superior or at least comparable to other approaches, except for occurrences of SSI, which are less frequent following laparoscopic repairs [24]. In direct comparisons between onlay and Rives-Stoppa techniques for incisional hernia repair, the Rives-Stoppa method has demonstrated superiority over onlay approaches due to lower rates of drain usage, shorter hospital stays, and fewer postoperative complications such as seromas and surgical wound infections [25].

Disadvantages

The Rives-Stoppa repair technique presents several disadvantages, including its higher technical complexity, longer surgical duration, and the necessity for surgeons with extensive experience due to its intricacy [25]. In a comparative study assessing the onlay and Rives-Stoppa techniques for incisional hernia repair, it was noted that patients undergoing the onlay technique experienced prolonged abdominal drainage time, extended hospital stays, and a heightened incidence of seromas and surgical wound infections in comparison to those treated with the Rives-Stoppa technique [25]. Moreover, in the context of complex ventral incisional hernias treated with the Rives-Stoppa repair utilizing a Mersilene prosthesis, while the overall recurrence rate was low, there existed a risk of prosthetic infection necessitating mesh explantation, which could contribute to hernia recurrence [26]. This highlights a potential complication associated with using prosthetic materials in the Rives-Stoppa repair technique.

Outcomes and Success Rates

The outcomes and success rates of Rives-Stoppa repair, also known as the GPRVS, have yielded positive results, particularly in addressing complex inguinal hernias. This technique achieves anatomical and prosthetic repair, fortifying the myopectineal region and establishing bilateral anatomical reinforcement, a feat that traditional anterior inguinal and ventral hernia mesh repairs may not accomplish [18]. The Stoppa procedure is widely regarded as a safe repair for bilateral inguinal hernias, with noticeable improvements observed over time in terms of operative duration, hospital stay, and morbidity rates, consequently leading to minimal recurrence rates [27]. Acknowledging that the Stoppa procedure necessitates a learning period to attain optimal results is crucial. Nonetheless, it has significantly enhanced outcomes over time, characterized by reduced morbidity and shorter hospital stays [27].

Comparative analysis

Surgical Approach and Incision Type

The surgical approach and incision type for inguinal hernia repair can differ between open and laparoscopic procedures. In open surgery, the repair is typically performed under local or regional anesthesia, with a single incision directly over the hernia. The surgeon then places the herniated tissue back into the abdomen and reinforces the weakened area with mesh, which aids in preventing recurrence [7,10]. Conversely, laparoscopic surgery involves making small incisions in the abdomen and utilizing a camera and specialized instruments to repair the hernia. This technique offers several advantages, including reduced postoperative pain, smaller incisions, quicker recovery, and lower recurrence rates than open surgery [10]. The decision between open and laparoscopic techniques depends on various factors, such as the patient's overall health, the surgeon's experience, and specific hernia characteristics. Laparoscopic repair may be preferred for recurrent or bilateral hernias, whereas open repair is often utilized for primary single-sided hernias. Both approaches have been endorsed as safe and effective by the National Institute for Health and Care Excellence (NICE) [10].

Operative Time and Anesthesia Considerations

Operative time and anesthesia considerations in inguinal hernia repair are pivotal factors that significantly influence patient outcomes. Recent research suggests that utilizing local anesthesia for inguinal hernia repair can result in shorter operative durations, particularly for patients aged under 75 years, without compromising safety or escalating complications [28,29]. Local anesthesia has been linked with decreased postoperative complications and swifter recovery room stays, rendering it a favorable option for older patients and potentially leading to substantial cost savings on a national scale [28,29]. Moreover, a comparative analysis between spinal and general anesthesia techniques for inguinal hernia repair underscored the advantages of utilizing the laryngeal mask airway in conjunction with propofol. This combination achieved shorter operative and recovery room durations, ensuring a low-risk and rapid recovery for patients undergoing this prevalent surgical procedure [30]. The selection of the anesthesia technique, whether local, regional, or general, hinges on various factors such as the patient's health status, surgeon preferences, procedure complexity, and anticipated duration, with each method offering distinct advantages and considerations [30,31]. The choice of anesthesia modality plays a pivotal role in both the operative time and postoperative outcomes of inguinal hernia repair. Local anesthesia holds promise in mitigating complications and expediting recovery times, particularly among older patients. Meanwhile, techniques like the laryngeal mask airway and propofol provide efficient and safe alternatives for achieving swift recovery in inguinal hernia surgeries [28-30].

Complication Rates

Complication rates vary between unilateral and bilateral inguinal hernia repairs. A comparative study between the two revealed complication rates of 11.2% for unilateral repairs and 16.5% for bilateral repairs, with no significant difference observed between the groups [32]. However, another investigation highlighted a higher incidence of postoperative complications within 30 days following bilateral repairs (4.9%) in contrast to unilateral repairs (3.9%), indicating a significantly greater risk associated with bilateral inguinal hernia repair [33]. This heightened risk was notably evident in the necessity for reoperation following bilateral repairs compared to unilateral repairs [33]. Furthermore, laparoscopic inguinal hernia repair has exhibited favorable outcomes with low complication rates, including a recurrence rate of 0.2% and a reoperation rate of 0.5%, with hematoma being the most common postoperative complication at 3.1% [34]. Studies have indicated that laparoscopic techniques offer advantages such as reduced pain, earlier return to activities, and lower recurrence rates than traditional anterior repair methods [34].

Postoperative Pain and Recovery

Postoperative pain and recovery following hernia surgery can vary in both intensity and duration. Acute postoperative pain is a common occurrence, but it can be effectively managed through a variety of techniques. These include using cold or heat packs, gently applying pressure with a pillow against the incision site, engaging in distraction methods such as playing games or listening to music, practicing relaxation techniques, and ensuring comfort measures such as adjusting room temperature and minimizing noise and light levels [35]. It's essential to adhere to prescribed pain medication regimens to ensure comfort and aid in the healing process [35]. However, in some cases, chronic postoperative pain can develop after hernia surgery, impacting mobility and quality of life. This type of pain may persist for months or even years and can be challenging to manage. Chronic pain following hernia repair is a recognized complication, with up to 16% of patients experiencing it after groin hernia repair [36]. Treatment strategies for chronic postoperative pain may involve a stepwise approach, beginning with watchful waiting and the use of systemic painkillers, escalating to nerve blocks, and considering surgery as a last resort, which may entail mesh removal and neurectomy in some instances [36]. Furthermore, specific techniques, such as the Rives-Stoppa repair for complex inguinal hernias, have demonstrated effectiveness in reducing postoperative pain and promoting better outcomes with minimal recurrence rates and reduced morbidity [37]. Understanding the different types of pain that can occur post-surgery, implementing appropriate pain management strategies, and seeking expert guidance for chronic pain issues are all crucial aspects of the recovery process after hernia surgery.

Long-Term Outcomes and Recurrence Rates

Long-term outcomes and recurrence rates in inguinal hernia repair indicate comparable recurrence rates in the long term for both open and endoscopic mesh repairs, as confirmed by data from the European Hernia Society guidelines [38]. The Rives-Stoppa repair remains the treatment for complex hernias, offering a suture-tension-free method with minimal recurrence rates and physiological healing processes [38]. Various studies comparing techniques, such as TEP, TAPP, and Lichtenstein repair, have shown differing outcomes regarding operative time, postoperative pain, analgesic requirement, and time to return to normal activities. However, similar recurrence rates after five years have been reported, ranging from 18% to 19% [38]. Additionally, a five-year prospective follow-up study of laparoscopic extraperitoneal (TEP) hernia repair demonstrated a favorable recurrence rate compared to open mesh repair, highlighting the long-term success of TEP hernia repair in achieving low recurrence rates [39]. Another study comparing laparoscopic TAPP and open preperitoneal (PP) repair found a relatively lower three-year recurrence rate after bilateral Lichtenstein repair than the Lap TAPP and open PP group [39]. Furthermore, research comparing the PP laparoscopic approach to the Stoppa operation indicated similar long-term recurrence rates between the two techniques [40].

Factors influencing technique selection

Patient-Specific Factors

Patient-specific factors are critical in determining outcomes and influencing the selection of hernia repair techniques. Modifiable risk factors such as obesity, diabetes, unhealthy alcohol use, and smoking have been identified as critical elements that can impact postoperative recovery and healthcare spending in ventral and incisional hernia repair (VIHR) [41,42]. These factors are linked to adverse outcomes following surgery, underscoring the importance of preoperatively optimizing them to enhance patient outcomes and reduce healthcare costs [42]. Moreover, patient-specific risk factors captured in surgical databases, including comorbidities, hernia characteristics, and wound characteristics, are vital for evaluating perioperative complications and optimizing patients before abdominal wall reconstruction procedures [41,43]. Preoperative patient assessment involves identifying modifiable risk factors across patient, hernia, and wound categories to optimize patients before reconstruction planning [41].

Surgeon Expertise and Preference

Surgeon expertise and preference significantly influence the approach for inguinal hernia repair. A study involving 21 practicing surgeons revealed that preference and autonomy, access to resources, and patient characteristics are key factors influencing the surgical approach used for inguinal hernia repair [44]. Surgeon preference and expertise may sometimes result in deviations from evidence-based guidelines, as evidenced by only 42% of surgeons opting for a minimally invasive approach to bilateral or recurrent inguinal hernias despite recommendations for a tailored approach based on individual patient factors [45]. Patients seeking a surgeon for hernia repair should consider various factors to ensure optimal care. These factors include specialization in hernia surgery, board certification, commitment to patient-centered care, proficiency in surgical techniques and approaches, experience with complex cases, provision of thorough follow-up care, communication style, hospital affiliation, and insurance coverage [46]. Surgeons with extensive experience, a proven track record of successful surgeries, and a dedication to ongoing education and training are likelier to deliver high-quality care, foster trust, and establish robust surgeon-patient relationships [46].

Hospital Resources and Infrastructure

Hospital resources and infrastructure play a pivotal role in the success of surgical procedures, including hernia repair. Factors such as bed capacity, including intensive care unit (ICU) beds, inpatient rehabilitation beds, skilled nursing beds, and the nurse-to-bed ratio, significantly influence the outcomes of these procedures [47]. Hospitals that have effectively addressed challenges like the weekend effect (WE) in hernia repair typically boast a higher mean number of inpatient rehabilitation beds (9.3 vs. 5.9) and a higher nurse-to-bed ratio (1.3 vs. 1.1) compared to facilities experiencing persistent WE [47]. Beyond bed capacity and staffing, hospital resources and infrastructure encompass surgical facilities and personnel, along with the availability of essential equipment and supplies [48]. Tools such as the Infrastructure, Procedures, Equipment, and Supplies (PIPES) tool and the WHO Surgical Safety Checklist have played vital roles in enhancing surgical outcomes, particularly in low- and middle-income countries [49]. Furthermore, specialized human resources and infrastructure are indispensable for advanced surgical techniques, such as robotic surgery, which can broaden the accessibility of minimally invasive procedures for hernia repair to a broader range of patients [50]. Notably, institutions like the Columbia Hernia Center have established dedicated research infrastructures focused on hernia care, positioning themselves at the forefront of innovation in abdominal wall surgery and contributing to developing new fields and techniques [50].

Cost Considerations

**Figure 1:**
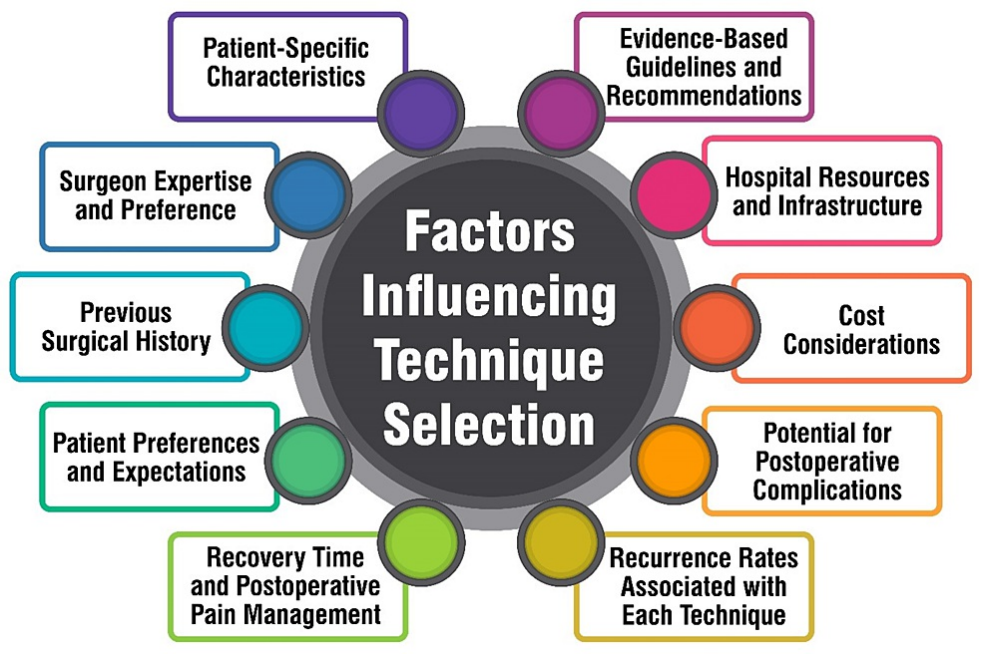
Factors influencing technique selection. Image credit: Sanjeev G. Gianchandani Gyani.

Cost considerations are paramount in the selection of hernia repair techniques. Research indicates that preoperative optimization can yield significant savings by mitigating complications and reducing readmissions following VIHR [42]. For instance, a 25% reduction in severe complications after VIHR could save a median of approximately $3.6 million, while a similar decrease in 30-day readmissions could result in nearly $6 million [42]. Furthermore, the laparoscopic approach for inguinal hernia repair typically carries slightly higher costs than open procedures, with bilateral repairs incurring higher expenses than unilateral ones [51]. Emergency operations for incarcerated inguinal hernias significantly escalate costs compared to elective surgeries [51]. Additionally, postoperative complications and subsequent reoperations exert a considerable cost-increasing impact on hernia repair procedures [51]. Various factors such as older age, multimorbidity, emergency operations, prolonged hospital stays, and postoperative complications are identified as significant drivers of costs in hernia repair [51]. Understanding these cost dynamics is vital for healthcare providers and policymakers to optimize resource allocation and enhance the cost-effectiveness of hernia repair procedures. Figure 1 shows factors influencing technique selection.

## Conclusions

In conclusion, the comparative analysis of bilateral open inguinal hernia repair and Rives-Stoppa repair techniques has provided valuable insights into managing bilateral inguinal hernias. Both approaches have distinct advantages and disadvantages, influencing operative time, postoperative pain, and recurrence rates. While bilateral open inguinal hernia repair offers simplicity and familiarity, Rives-Stoppa repair may offer advantages regarding reduced recurrence rates and postoperative complications. These findings have significant implications for clinical practice, highlighting the importance of considering patient-specific factors, surgeon expertise, and hospital resources when selecting the optimal surgical approach. However, further research is needed to conduct long-term comparative studies evaluating outcomes beyond recurrence rates and exploring surgical techniques and materials innovations. Such research endeavors hold the potential to improve patient outcomes, reduce healthcare costs, and advance the field of inguinal hernia repair.
